# Enhanced Multiple Instance Representation Using Time-Frequency Atoms in Motor Imagery Classification

**DOI:** 10.3389/fnins.2020.00155

**Published:** 2020-02-25

**Authors:** Diego Collazos-Huertas, Julian Caicedo-Acosta, German A. Castaño-Duque, Carlos D. Acosta-Medina

**Affiliations:** ^1^Signal Processing and Recognition Group, Manizales, Colombia; ^2^Cultura de la Calidad en la Educación Research Group, Universidad Nacional de Colombia, Manizales, Colombia

**Keywords:** motor imagery, LASSO regularization, CSP, multiple-instance learning, dynamic brain behavior

## Abstract

Selection of the time-window mainly affects the effectiveness of piecewise feature extraction procedures. We present an enhanced bag-of-patterns representation that allows capturing the higher-level structures of brain dynamics within a wide window range. So, we introduce augmented instance representations with extended window lengths for the short-time Common Spatial Pattern algorithm. Based on multiple-instance learning, the relevant bag-of-patterns are selected by a sparse regression to feed a bag classifier. The proposed higher-level structure representation promotes two contributions: (i) accuracy improvement of bi-conditional tasks, (ii) A better understanding of dynamic brain behavior through the learned sparse regression fits. Using a support vector machine classifier, the achieved performance on a public motor imagery dataset (left-hand and right-hand tasks) shows that the proposed framework performs very competitive results, providing robustness to the time variation of electroencephalography recordings and favoring the class separability.

## 1. Introduction

Motor Imagery (MI), recorded via electroencephalography (EEG), is the motor intention in the form of enhancement or attenuation of brain activity in μ and β rhythms over the sensorimotor cortex, having a different spatial distribution for each imagined movement (Liang et al., 2016). MI has particular applicability to neurophysical regulation and rehabilitation, unconscious motor preparation (Wang et al., 2019), games and entertainment, and sports training, among others (Suica et al., 2018). In education scenarios, for which the Media and Information Literacy methodology proposed by the UNESCO covers several competencies that are vital for people to be effectively engaged in all aspects of human development (Frau-Meigs, 2007). In this regard, the usage of modern technologies like motor imagery learning may provide tools for measuring the time response of brain activations during imagination of a determined movement, helping to assess the sensorimotor response-ability under very concrete learning stimuli (Guillot and Debarnot, 2019). Nonetheless, in characterizing these event-related potentials (ERP), a significant drawback is the intrinsic variations between individuals and within individual trials, making the EEG energy distribution very dependent on time, frequency, and spatial domains (Yamawaki et al., 2006). Due to this heterogeneity, therefore, finding spatial patterns of neural activation with maximal class separation is a challenging issue and may require a tiresome process (Padfield et al., 2019).

For bi-conditional tasks, Common Spatial Pattern (CSP) filtering remains the most widely used signal projection technique to extract discriminative EEG features, maximizing the variance of one condition while minimizing the alternative one. Still, the effectiveness of CSP is mainly affected by two aspects (Ang et al., 2012): Low robustness of selected channels across trials, and selection of the time-window for piecewise analysis that must cover the interval within a neural pattern is activated, but removing the unrelated sampling points (Miao et al., 2017). However, due to the subject-specific nature of MI tasks, the use of a single time window for a whole subject population diminishes the discriminating ability to extract CSP features and, hence, degrading the classification accuracy (Xu et al., 2015). To improve robustness at low Signal-to-Noise Ratio (SNR), optimal frequency band selection is a common approach that exploits the spectral relationship among the extracted MI features. Namely, sparse regression is applied to find compact representations within a supervised learning framework, determining the significant CSP features with class labels (Zhang et al., 2015). Nonetheless, the enhancement of sparse CSP-based systems should consider the design of an optimized dictionary matrix utilizing subject-adapted frequency-time patterns for increasing the classification accuracy (Shin et al., 2012).

For extracting feature dynamics, the search for adequate time-series similarity is given close attention, centering mainly on finding shape-based relationships. To this end, the point-to-point comparison is the most implemented algorithm, being efficient for short-time series or with periodic waveforms to extract local temporal and/or frequency information. In the case of long sequences, instead, it is more appropriate to consider similarities based on higher-level structures like the bag-of-patterns representations, capturing more accurately the structural dynamics to be fed into a classifier and without having to deal with the raw data (Passalis et al., 2017). Besides, despite ignoring the temporal order of local segments, theses representations enable capturing high-level structural information, relating either local and/or global relationships (Wang et al., 2013). To create bag-of-patterns representations (Chen et al., 2006), similarity distances between sets are used as well as similarity functions between bags and instances (Cheplygina et al., 2015), extracted from histograms (Lin and Li, 2009), codebooks (Gui and Yeh, 2014), or multivariate bag-of-words models (Bailly et al., 2016). Consequently, the construction of bag-of-patterns and selection of a similarity measure are the main aspects to implement a structural relationship between time series, reducing the similarity bias effect because of the presence of outliers in real-world applications and avoiding high dimensional feature vectors that may limit their application for large datasets (Ratanamahatana and Keogh, 2004).

With the aim of improving the distinction of brain dynamics in motor imagery tasks, we propose an enhanced bag-of-patterns representation that is developed through Multiple Instance Representation using Time-Frequency Atoms. In this case, instances are constructed by CSP feature sets extracted over short time windows of the filtered EEG signal. To explore the brain dynamics across time in more detail, augmented instance representations are introduced, which are obtained by the scale and shift operations over the original piecewise CSP feature extraction. Using a multiple-instance learning approach, the relevant bag-of-patterns are constructed and further selected by LASSO regression. From obtained weight vector by LASSO, the brain dynamic is modeled as the weighted appearance probability of each time instant along MI period, highlighting the more relevant time intervals associated with the response variable. The performance of proposed enhanced bag-of-patterns representation is assessed using a Support Vector Machine (SVM) classifier on a public dataset (left hand and right hand MI tasks), showing that the accomplished accuracy is very competitive, providing robustness to the time variation of EEG recordings and favoring the class separability.

The agenda is as follows: Firstly, section 2 describes the mathematical background of CSP feature extraction, optimized multi-instance learning representation, and pattern selection by LASSO regularization for MI classification. The developed experiments and achieved results are described in section 3, respectively. Finally, the discussion and concluding remarks are provided in section 4.

## 2. Materials and Methods

### 2.1. Motor Imagery Dataset 2a

This signal collection, publicly available at^1^, was recorded from nine subjects using a 22-channel system (with inter-electrode distances of 3.5 cm), corresponding to the international 10−20 system. The EEG trials were recorded at sampling frequency 250 Hz and regarded one of four MI tasks: left hand, right hand, both feet, and tongue. All recordings were performed in six runs separated by short breaks so that each run held 48 trials (each one lasting 7 s). A short beep indicated the trial start, after which a fixation cross appeared on the black screen within the first 2 s. Then, an arrow (or cue) was shown during 1.25 s to indicate the left, right, up, or down directions, stimulating to imagine a left hand, right hand, both feet, or tongue movement, respectively. Next, each subject performed one MI task within the time interval from 3.25 to 6 s, while waiting for the cross to reappear again. The EEG data were labeled (*l*∈{+1, −1}, left hand or right hand, respectively) and the artifacts removed.

For preprocessing, the raw EEG data are band-pass filtered using *N*_*f*_ overlapped bandwidths, resulting in a matrix $X_{rf}^{l}={\left[x_{rf}^{c}:f\in N_{f},r\in R\right]}^{T}$, where the vector $x_{rf}^{c}\in {\mathbb{R}}^{⊤}$ denotes each filtered channel *c* per bandwidth *f* and trial *r*. Specifically, we use *R* = 144 and *N*_*f*_ = 17 five-order overlapped Butterworth filters with bandpass frequencies between 4 and 40 Hz, having a bandwidth of 4 Hz and overlapping rate of 2 Hz as in Zhang et al. (2015). Then, the attained filter-banked signals are time-windowed onto *N*_*t*_ intervals with a 90% overlap of samples, each one lasting *t*.

As a result, the processing stage provides an input *time-frequency* matrix $X_{rft}^{l}={\left[x_{rft}^{c}:t\in N_{t}\right]}^{⊤}$ per trial, where $x_{rft}^{c}\in {\mathbb{R}}^{T}$ represents the time-segmented neural activity related to motor imagery within a concrete bandwidth, measured at each channel, lasting *T* *s*.

### 2.2. Construction of *Time-Frequency* CSP-Based Atoms

Given a labeled trial matrix $X_{rft}^{l}\in {\mathbb{R}}^{T\times C},$ the algorithm of Common Spatial Patterns aims at finding, within each channel *c*⊂*C*, the linear transformation vector ***w*** ∈ ℝ^*C*^ to maximize the Rayleigh Quotient (RQ, noted as *J* ∈ℝ^+^) that is computed through the mapped data variance between classes as follows:

(1) $$\begin{array}{r} w^{*}=\underset{\forall w}{\text{max}}J\left(w\right)=\frac{w^{⊤}\Sigma^{\left(+1\right)}w}{w^{⊤}\Sigma^{\left(-1\right)}w},\;\text{s}.\text{t}.:||w|{|}_{2}=1 \end{array}$$

where $\Sigma^{\left(l\right)}=E\left\{X_{rft}^{l}X_{rft}^{l⊤}:\forall r\in R\right\}$, with **Σ**^(*l*)^ ∈ℝ^*C*×*C*^, is the simplest estimate of class data variance computed at a frequency *f*. Notations ||·||_*p*_ and *E*{·:∀*r*} stand for ℓ_*p*_-norm and expectation operator across variable *r*, respectively.

Further, the unlabeled EEG sample ***X***_*rft*_ is filtered through the learned spatial matrix ***W*** ∈ ℝ^*C*′^ × *C* that holds all *C*′ piecewise transformation components. Thus, at each time sample *t* ∈ *N*_*t*_, the projected data ***Z***_*rft*_ = ***WX***_*rft*_, with $Z_{rft}\in {\mathbb{R}}^{C^{\prime }\times T},$ is computed by only 2*M* representative terms of *C*′ (namely, *M* first and *M* last rows), yielding the extracted feature vector ***d***_*rt*_ that is calculated across the spectral domain as below:

(2) $$\begin{array}{l} d_{rt}=\ln\;\left(\text{diag}\left(\text{var}\left\{Z_{rft}\right\}\right)/\mathrm{tr}\left\{Z_{rf_{1}t}\right\}\right)||\cdots \\ \;||\ln\;\left(\text{diag}\left(\text{var}\left\{Z_{rf_{N_{f}}t}\right\}\right)/\mathrm{tr}\left\{Z_{rf_{N_{f}}t}\right\}\right) \end{array}$$

where var{·} and tr{·} denote the variance and trace operator, respectively.

Using the extracted feature from each trial *r* and overlapped time window *t*, therefore, we obtain the time-frequency feature array $D_{r}\in {\mathbb{R}}^{2MN_{f}\times N_{t}}$ (*N*_*t*_ < 2*MN*_*f*_) with vector columns $d_{rt}\in {\mathbb{R}}^{2MN_{f}},$ hereafter we will term as *time-frequency atoms*.

### 2.3. Optimization of *t-f* Atoms for MIL Representation

Relying on the above introduced atoms, we define each column vector ***d***_*rt*_ as an *instance* within a Multi-Instance Learning (MIL) framework, and in turn, we assemble each *r*-th *bag* per trial by arranging the whole column in a vector $B_{r}=\left[\begin{array}{c} d_{r1}^{⊤}\\ \ldots \\ d_{rN_{t}}^{⊤} \end{array}\right]$with $B_{r}\in {\mathbb{R}}^{N_{t}\times 2MN_{f}}$. So, the whole training set, extracted over all trials, is denoted as $B=\left\{B_{r}:\forall r\in 2R\right\}$.

To put into effect the *concept* that makes a bag label either positive or negative, the instance-based embedding is intended to map each bag into a vector space. To this end, we specify each *k* concept class, noted as ***d***^*k*^, through the single point concept class, assuming as a target concept each instance extracted in Equation (2), i.e., $d^{k}\subset D=\left\{d_{rt}:\forall r,t\right\}$.

For improving the target concept set, the CSP feature set extracted by Equation (2) is further refined within each instance in terms of contributing to distinguishing between the labels. In particular, we search the relevant information from each EEG channel through a sparse-based operator over the CSP feature space calculated at each time window. As suggested in Caicedo-Acosta et al. (2019), the Multiple-Instance Logistic Regression estimates a bag label by aggregating each instance label through the following Bernoulli-based logistic regression ${\tilde{l}}_{r}=I\left(\underset{t\in N_{t}}{\sum }Bernoulli\left(h\left(\beta_{0}+d_{rt}^{⊤}\beta \right)\right)>0\right)$, where I(x)=1 if *x*>0, otherwise, I(x)=0, being *h*(*x*) = 1/(1+*e*^−*x*^) the sigmoid units.

In particular, the parameter set (one bias term β_0_ ∈ ℝ and $\beta \in {\mathbb{R}}^{2MN_{f}}$) is computed within the following quadratic optimization framework (Chen et al., 2017):

(3) $$\begin{array}{l} \underset{\beta_{0},\beta }{\text{min}}(-\underset{r\in 2R,t\in N_{t}}{\sum }l_{r}\gamma_{rt}^{i}(\beta_{0}+d_{rt}^{⊤}\beta )\\ -\text{log}(1+\text{exp}\left(\beta_{0}+d_{rt}^{⊤}\beta \right))+\alpha \underset{q\in 2MN_{f}}{\sum }|\beta_{q}|) \end{array}$$

where the conditional expectation (given *l*_*r*_ = 1) is computed as $\gamma_{rt}=h\left(\beta_{0}+d_{rt}^{⊤}\beta \right)/\left(1-\prod_{t=1}^{N_{t}}1-h\left(\beta_{0}+d_{rt}^{⊤}\beta \right)\right)$ and α ∈ ℝ^+^ is a regularization term. Here, we solve the above optimization problem through an iterative coordinate descent algorithm.

As a consequence, by applying the optimizing procedure on MIL representation, we select the *t-f* atom set with improved properties, noted as ${\hat{d}}_{rt}$, since it holds in Equation (3) that the larger the magnitude of β_*q*_ – the more relevant the feature to predict the instance labels. Therefore, the performed atom-based optimization enhances the MIL representation, allowing discarding irrelevant or redundant information in discrimination MI tasks.

### 2.4. Similarity-Based Enhancement of Expanded MIL Representation

With the aim of implementing the *concept* introduced before, the instance-based embedding is intended to map each bag into a vector space. As proposed in Chen et al. (2006), the feature vector $p_{r}\in \mathbb{R}{\left[0,1\right]}^{2R}$ extracted from each bag is computed by the conditional probability that *k* instance belongs to *r*-bag, $P\left({\hat{d}}^{k}|B_{r}\right)$, as follows:

(4) $$\begin{array}{r} p_{r}=\left[P\left({\hat{d}}^{k}|B_{r}\right):\forall {\hat{d}}^{k}\in D,k\in 2RN_{t}\right]. \end{array}$$

Further, to implement the higher-level time-frequency representation, we accept the mapping of instance-based features with both multiple concepts, either positive or negative, which are extracted from the training dataset, D⊂B, so that the conditional probabilities in Equation (4) can be approximated as:

(5) $$\begin{array}{r} s\left({\hat{d}}^{k},B_{r}\right)=P\left({\hat{d}}^{k}|B_{r}\right)\propto \underset{\forall t\in N_{t}}{\text{max}}\text{exp}(-||{\hat{d}}_{rt}-{\hat{d}}^{k}|{|}^{2}/\sigma^{2}) \end{array}$$

where $s\left({\hat{d}}^{k},B_{r}\right)\in \mathbb{R}\left[0,1\right]$ is the assessed measure of similarity between concept ${\hat{d}}^{k}$ and bag ***B***_*r*_, while σ ∈ ℝ^+^ is the bandwidth of exponential square function. Using the measure in Equation (5), therefore, we perform the similarity matrix $S\in \mathbb{R}{\left[0,1\right]}^{2RN_{t}\times 2R}$ with elements $s\left({\hat{d}}^{k},B_{r}\right),$ ∀*k* ∈ 2*RN*_*t*_, ∀*r*∈ 2*R*.

Intending to include the mutual influence of other atoms extracted at different time instants, we expand further each similarity representation in Equation (5) by a time-window scale, Δτ, yielding the following enhanced similarity matrix:

(6) $$\begin{array}{r} \Delta_{pq}=\left[S_{p\Delta \tau }||\cdots ||S_{N_{\tau }\Delta \tau }:\forall p,q\right], \end{array}$$

where $S_{\tau }=\left[s\left({\hat{d}}_{\tau }^{k},B_{r,\tau }\right):\forall r,k\right]$ is an version of ***S*** with elements expanded by a multiple scaling factor τ = *pΔτ*, *p* = [*q, N*_τ_], *q* = [2, *N*_τ_], being *N*_τ_ the maximal number of considered time-window lengths τ.

Further, as a powerful preprocessing step for high-dimensional data analysis, feature selection is carried out aiming at discarding irrelevant and redundant features from the expanded similarity representation $\Delta_{pq}\in \mathbb{R}{\left[0,1\right]}^{2R\times \left(N_{t}\left(N_{\tau }-q\right)\right)}$ using the LASSO sparse regression model that has been shown to be efficient in MI tasks within the following optimizing framework (Feng et al., 2019):

(7) $$\begin{array}{r} u^{*}=\arg\underset{u}{\text{min}}||\Delta_{pq}u-l|{|}_{2}^{2}+\lambda ||u|{|}_{1} \end{array}$$

where ***l*** ∈ ℝ^2*R*^ is a vector with the class labels, $u\in {\mathbb{R}}^{N_{t}\left(N_{\tau }-q\right)}$ is a sparse vector to be learned, λ ∈ ℝ^+^ is a positive regularization parameter for controlling the sparsity of ***u***. Lastly, a SVM-based algorithm is implemented on the reduced subset of selected features ${\hat{\Delta }}_{pq}$ to train the bag classifier. Algorithm 1 summarizes the similarity approach for MIL representation developed in this section.

**Algorithm 1:** Expanded similarity of MIL representation

Ensure: Optimal bag formed by atom combination from instances having a window-size vector ${\hat{\Delta }}_{pq}$
1: **Δ**_*pq*_ = ϕ
2: **for** *q* = [τ_*init*_, *N*_τ_] **do**
3: **for** *p* = [*q, N*_τ_] **do**
4: $S_{\tau }=\left[s\left({\hat{d}}_{\tau }^{k},B_{r,\tau }\right):\forall r,k\right]$ Calculate similarity matrix
5: Δ_*pq*_ ← ***S***_*pΔτ*_
6: ${\tilde{l}}_{i}^{pq}=\text{SVM}\left(\Delta_{pq}\right)$
7: ${\hat{\Delta }}_{pq}={\text{arg max}}_{\forall pq}E\left\{l_{i}-{\tilde{l}}_{i}^{pq}E\right\}$

## 3. Experimental Evaluation

**Figure 1:**
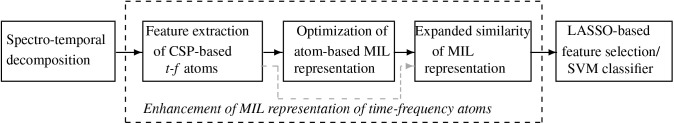
Scheme of bag-of-patterns representation proposed for classification of bi-class motor imagery tasks. Within the MIL framework using *t-f* atoms, the suggested improvement is remarked by a dashed box.

As illustrated in Figure 1, the proposed enhancement of bag-level representation employs a CSP-based feature set, and appraises the following procedures: (i) Frequency-temporal decomposition of EEG raw data; (ii) Feature extraction of *t-f* atoms through a CSP approach, (iii) Optimization of *t-f* atoms by Multiple-Instance Logistic Regression; (iv) Extension of similarity assessments for MIL representation over a wider domain; and (v) Feature selection performed by LASSO strategy, feeding a classifier of MI bi-class tasks. Intending to evaluate the contribution of atom optimization, we consider two scenarios of training: including this stage and without it (gray line in Figure 1).

### 3.1. Experimental Set-Up

The experiment is performed for each subject, for which approximately 138 trials per subject are presented. For validation purposes, the feature set is extracted from EEG data using the algorithm of Common Spatial Patterns, for which the filter dimension is adjusted to *M* = 3, corresponding to the more representative terms of *C*′ as suggested in Blankertz et al. (2008). Besides, we employ classification accuracy as the performance criterion that is estimated by a support vector machine algorithm through a 10-fold cross-validation scheme. In the proposed expanded representation stage, the investigated range of τ is adjusted to discrete values [0.2−2.0] s to embrace the whole motor imagery period, while the slicing Δτ is empirically adjusted to 100 ms. In Table 1, we report the total number of instances vectors according to τ value. Moreover, in classification stage, the SVM kernel bandwidth and LASSO regularization coefficients were fixed through an exhaustive search within a range of [10^−3^, 10^−2^, 10^−1^, 10^0^, 10^1^, 10^2^, 10^3^] and [10^−10^−9^−1^] in a logarithmic scale, respectively.

**Table 1:** Amount of instances performed by each tested time-window.

τ[*s*]	0.2	0.3	0.4	0.5	0.6	0.7	0.8	0.9	1.0	1.1	1.2	1.3	1.4	1.5	1.6	1.7	1.8	1.9	2.0
*N*_*t*_	91	54	41	29	24	19	16	12	11	9	7	6	5	4	3	2	2	1	1
Σ_*N*_*t*__	91	145	185	215	239	258	274	286	297	306	313	319	324	328	331	333	335	336	337

*The last row reports the cumulative sum across all range of considered τ*.

### 3.2. Computation of CPS-Based *t-f* Atoms

Table 2 displays the optimal time window (heuristically determined), performing the highest accuracy for each subject as well as for the whole set. As a result, the optimal span of discrete τ ranges extensively from 0.2 till 2.0 s, implying that each subject differently rules all changes of *J* and, therefore, posing a difficulty in performing the group analysis across the whole subject set. Note that there is no dependence between the window length and achieved accuracy.

**Table 2:** The highest accuracy scores performed by each subject by fixing the optimal time window (τ^*^), and across the whole subject set (fixing τ = 2 s).

	**A08T**	**A09T**	**A03T**	**A01T**	**A05T**	**A07T**	**A06T**	**A04T**	**A02T**	**Average**	**Whole set**
τ	0.3	1.0	1.5	2.0	1.5	2.0	0.2	1.5	0.2		1.5
$a_{\tau^{*}}$	97.7 ± 3.2	100.0 ± 0.0	98.9 ± 2.5	93.7 ± 5.3	90.6 ± 4.9	94.8 ± 6.1	73.2 ± 7.0	65.3 ± 6.5	63.6 ± 5.4	86.4 ± 4.5	89.4 ± 4.7
*a*_τ = 2_	95.8 ± 3.3	97.3 ± 3.8	98.0 ± 2.6	93.7 ± 5.3	88.8 ± 4.6	94.8 ± 6.1	67.6 ± 16.4	64.0 ± 9.8	60.3 ± 10.6	84.5 ± 6.9	88.0 ± 5.2

**Figure 2:**
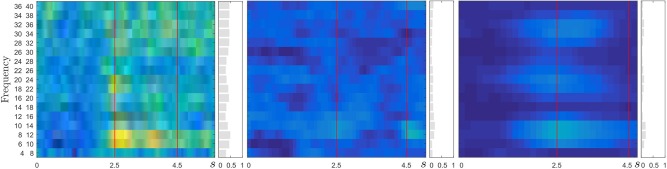
Estimation of *J* using the optimal time window τ. **(Left)** the subject A08 (achieving the best accuracy), **(Center)** the patient A02 (worst accuracy), and **(Right)** the group analysis performed at the admitted value τ = 2 s for validating the tested MI Dataset 2a. Spectral relevance is colored in gray bars.

For the sake of illustration, Figure 2 represents the CSP cost function value *J* (i.e., RQ value) that is estimated over frequency domain at each optimal time window τ. As seen in the left plot, the subject marked as A08 performs the best at a small window τ = 0.3 s, meaning that the rapid dynamics are relevant in differentiating between classes. Instead, the worst case (A02) demands τ = 0.2 s, that is, the relevant dynamics are also fast, but widely scattered over time as observed in the center plot. Therefore, the choice of time window remains crucial to characterize the changes in neural activity through the baseline atom representation.

Another aspect is the spectral relevance that is assessed as the marginal values of *J* on the frequency bands (bars painted in gray color). Even that *left* and *center* plots in Figure 2, indicate that the optimal frequency bands are subject-specific, the *right* plot shows the Rayleigh quotient of the whole subject set is mostly localized within μ (8–13 Hz) and β (13–25 Hz) rhythms as widely-accepted. The applied group analysis performs concatenation of all single-subject data into a single group array, i.e., ${⋃}_{\forall m}X_{m}^{l}$ from which a latent structure of sources is computed (Padilla-Buritica et al., 2019).

### 3.3. Performance of MIL Representation Using *t-f* Atoms

Using the enhanced representation in Equation (6), we validate the present proposal in discriminating against the bi-task MI dynamics. Thus, using the most-likely-cause estimator in Equation (5) as the similarity metric, we investigate the influence of τ (the relationship window between time series) on the produced *t-f* dynamics that are the most relevant in discriminating between classes.

**Figure 3:**
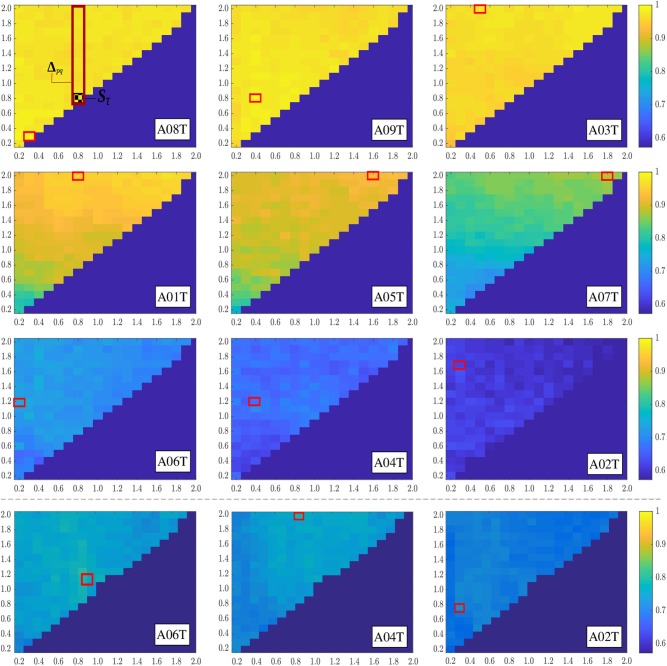
Accuracy performed at different window length combinations of atom-based instances. The last row beneath the dotted line displays the subject performance with a lower accuracy (A06T, A04T, and A02T) after using the optimization of the atom-based MIL representation stage.

Figure 3 display the accuracy achieved by estimating the higher-level structure similarity for each one of the available combinations of atom-based instances of time-frequency representation, that is, ***S***_τ_, and **Δ**_*pq*_, for which the graphical meaning is drawn to get a better understanding of the proposed dictionary expansion. The red-box represents the expansion that achieves the best performance in each subject. Due to the symmetry of matrix accuracy in τ, only its upper part is reported and ranked in decreasing order of accuracy achieved by each subject. Namely, the *x* axis represents the considered time-window sizes, while *y* axis shows all possible expansions, that is, the different combinations of time-window sizes present in an expanded bag. Thus, the diagonal of the matrix represents the bags composed of instances formed from a single-time-window size. Besides, the last row of the image represents the performance of the three subjects with the lowest performance using the bag optimization stage.

**Figure 4:**
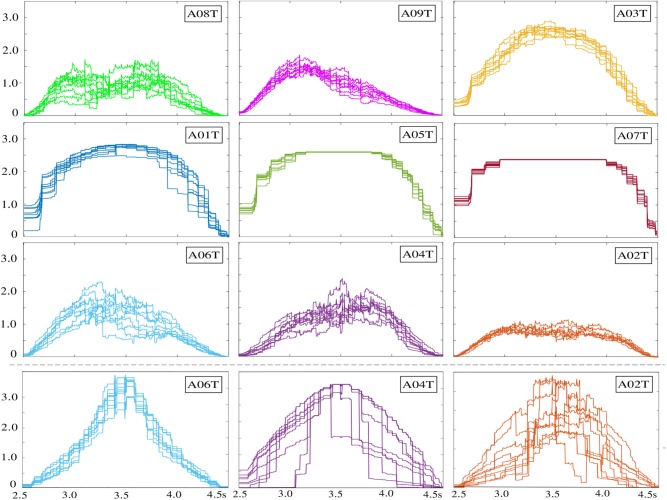
Temporal dynamics from the absolute LASSO weights performed within the motor imagery period. Each time series is a cross-validated fold. The last row displays the subjects with lower accuracy after the optimization of the atom-based MIL representation stage.

In terms of distinguishing between MI tasks, the measured similarity matrix values allow extracting the relevant dynamics from expanded MIL representations, facilitating an accuracy enhancement over a wide range of τ. Further, we rely on the LASSO fits estimated by the feature selection task in Equation (7), which increases the model interpretability by eliminating irrelevant variables that are not associated with the response variable and this way also reducing the overfitting (Roth, 2004; Fonti and Belitser, 2017). Namely, besides information about feature relevance, some indication is given about the degree up to which a feature is relevant or can be replaced by others. Nevertheless, the solutions tend to be not consistent estimations of the underlying “true” weight vector ***u***, regarding its exact value as quoted in Pfannschmidt et al. (2019). As shown in the Figure 4, therefore, we compute the normalized absolute LASSO weights at each time instant, but including the estimation performed for 10 cross-validated folds. It is worth noting that computation of LASSO fits directly through the Rayleigh quotient in Figure 2 does not provide an understandable representation of brain dynamics at each time instant since the optimal vector u^*^ holds the extracted CSP features, but contributing across the whole MI period. Instead, the learned sparse vector from the bag-of-patterns representations reveals a dynamic behavior that somehow resembles an elicited ERP waveform, rising at the beginning and declining in the closing periods.

For subjects A08T, A09T, and A03T, the measured similarity values enable an accuracy that is high practically regardless of the examined window of atom-based instances, though each subject has a distinct dynamic behavior. In the case of subjects A01T, A05T, and A07T, their performance accuracy is a bit reduced, while the time range of optimal windows also shrinks. Moreover, the relevant dynamics of enhanced MIL representation are extracted by larger values of τ (close to 2 s) so that the LASSO fits remain constant over a large window of bag representation instances. In the last triad of subjects (A06T, A04T, and A02T), the relevant dynamics are scattered all over the range of considered short-time window τ, but appearing in a noticeably worse accuracy. Note that the dynamic learned by the sparse vector of A02T has low values. The last row in Figure 4 represent the rise of accuracy performed by optimizing the similarity of enhanced MIL representation through the procedure in section 2.4. As a result, the LASSO fits may increase prominently and remain constant over extensive window lengths.

**Figure 5:**
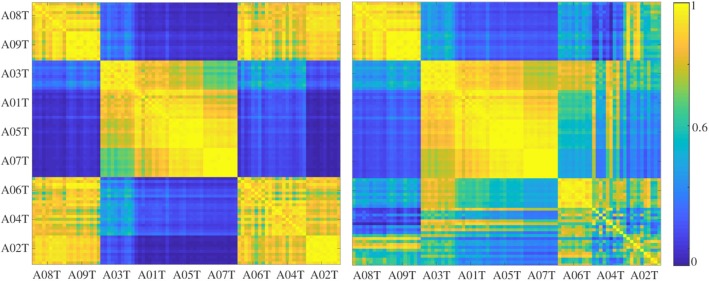
Pairwise similarity between subjects across the trial set assessed when omitting the optimization of *t-f* atoms **(Left)** and using the Multiple-Instance Logistic Regression **(Right)**.

To evaluate the influence of the used optimization procedure, we perform the pairwise similarity between subjects across the trial set. The left plot of Figure 5 presents the case when optimization is omitted, showing an apparent clustering of three groups of patients. However, the last triad of patients, achieving the worst accuracy, turns out to be very similar to the best triad, resulting in an inconsistency. In turn, the right plot degrades the similarity of the worst triad between subjects, as well as with other patients. Therefore, we may hypothesize that the use of Multiple-Instance Logistic Regression allows for improving the performed accuracy by better extracting the discriminating dynamics of MIL representations.

For the sake of comparison, we contrast the accuracy performance achieved the proposed enhancement of bag-level representation using a CSP-based feature set against the following three state-of-the-art approaches based on Filter-Bank preprocessing and CSP-based feature extraction procedure: (i) Sparse Filter Bank Common Spatial Patterns (SFBCSP) (Zhang et al., 2015) used as baseline approach, (ii) Spatial-Frequency-Temporal Optimized Feature Sparse Representation-based Classification(SFTOFSRC) (Miao et al., 2017), which includes a time-decomposition stage in the data preprocessing to generate a column vector of extracted CSP features for sparse representation-based classification, and (iii) Temporally Constrained Sparse Group Spatial Patterns(TSGSP) (Zhang et al., 2018) that adding a LASSO-based regularization term in the time domain. Table 3 displays the accuracy of the compared LASSO-based CSP algorithms reported for each subject, showing that all of them are outperformed by the proposed enhancement of bag-level representation using an SVM instance classifier, at least, in terms of the average across the whole subject set. Furthermore, by optimizing the higher-level structure similarity of bag-based representation, the performed accuracy increases further, improving most of the subjects that perform a low signal-to-noise ratio. Lastly, we evaluate the significance in terms of the disagreement of performing individual accuracy between the proposal and each of the comparison methods. To this end, the paired *t*-test is conducted at a fixed confidence value of *p* < 0.1, employing the scores achieved on the cross-validation folds (this information is not available for TSGSP). As seen in Table 3, there are confident differences of performance with the proposal (underlined subjects) for most of the individuals using SFTOFSRC. However, using SFBCSP, only a few subjects achieve a distinctive accuracy, probably because of the obtained higher dispersion of that approach.

**Table 3:** Comparison of SVM accuracy achieved by the proposed bag-level representation.

**Subject**	**TSGSP**	**SFBCSP**	**SFTOFSRC**	**Proposal**	Proposal*
A08T	95.8	97.0 ± 2.9	96.9 ± 3.4	99.0 ± 1.7	**99.2** **±** **1.6**
A09T	81.3	97.8 ± 3.1	94.6 ± 3.4	98.7 ± 2.1	97.3 ± 3.2
A03T	93.8	98.8 ± 1.7	98.5 ± 1.9	97.7 ± 2.6	**99.2** **±** **1.6**
A01T	87.0	91.8 ± 4.7	91.8 ± 3.9	**96.0** **±** **1.1**	94.8 ± 3.5
A05T	90.4	90.6 ± 3.7	95.7**±** **2.1**	93.0 ± 3.1	95.3 ± 4.4
A07T	91.4	94.7 ± 6.1	76.3 ± 5.7	88.0 ± 4.5	**96.2** **±** **4.6**
A06T	63.9	67.9 ± 6.9	71.0 ± 6.4	**74.5** **±** **4.8**	72.4 ± 7.9
A04T	**74.3**	63.5 ± 10.6	69.0 ± 7.1	70.3 ± 6.8	69.5 ± 9.3
A02T	64.7	58.4 ± 8.3	62.8 ± 5.9	64.0 ± 5.9	**66.2** **±** **5.3**
*Average*	82.5	84.5 ± 5.3	84.0 ± 4.4	86.8 ± 3.5	**87.8** **±** **4.6**

Each method performing the best individual accuracy is marked in bold. Abbreviation Proposal denotes the enhanced representation without optimizing the t-f atoms, while notation * includes this procedure. Each underlined subject achieves confident differences of performance with either proposal version.

## 4. Discussion and Concluding Remarks

Intending to extract patterns of the brain activity that allow improving the class separation, we propose the enhancement of bag-level representation using a CSP-based feature set. For this purpose, we exploit the baseline short-time CSP feature extraction, introducing an expanded atom-based MIL representation, covering more extensive window lengths. The obtained results in a public dataset prove that the accomplished accuracy is very competitive, providing robustness to the time variation of EEG recordings and favoring the class separability. Nevertheless, the following aspects for implementing our proposal are to be mentioned:

– *Construction of CSP-based t-f atoms*: As seen in Table 2 and Figure 2, there is not a clear relationship between the window length and achieved accuracy. These facts indicate that a frequency band decomposition of multi-channel EEG combined with an accurate time segmentation is necessary to improve the MI classification accuracy. The alone optimization of frequency bands without considering the influence of time may fail in finding the optimal feature set for classification (Miao et al., 2017). In addition, the obtained results show that the performance of the enhanced bag-level representation is influenced by the CSP-based representation, demanding high values of SNR for the acquired EEG data. This fact is evidenced enough by the subject A02T, whose performance is seriously diminished because of the low SNR.
– *Atom-based MIL representation*: With the aim to improve the contribution of *time-frequency* CSP-based atoms, we perform the Multiple-Instance Logistic Regression, that allows discarding irrelevant or redundant information in discrimination MI tasks. As a result, the performed accuracy importantly increases, as shown for the subjects achieving the worse outcomes without optimization. This improvement may be explained due to the LASSO fits increase prominently and remain constant over extensive window lengths.
– *Accurate similarity of expanded MIL representation*: The benefit of mapping the CSP feature space to an expanded bag representation is to disclose the data distribution, capturing/encoding more meaningful information (local and global).
– *Computational burden:* As regards the complexity of developed learning algorithms, however, it is so high that real-time processing during training does not apply. The learning procedure includes an exhaustive analysis within the parameter setting stage, which holds approximately 10*N*_τ_≈190 possible expanding configurations, where *N*_τ_ is the number of considered time windows τ. Table 4 summarizes the algorithmic complexity of compared approaches. As a result, in comparison with the baseline algorithm with no Multiple-Instance Learning, the computational burden of training procedures, for the tested database, rises as much as 1,500 times! Nevertheless, once the corresponding training procedures are performed, the validating classification algorithms of developed approaches may be suitable for real-time processing.

**Table 4:** Algorithm complexity of developed learning procedures.

	**Proposal**	Proposal^*^
Time [*h*]*^a^*	36	36*N*_λ_
Complexity	$O\left(n^{2}\right)$	$O\left(N_{\lambda }n^{2}\right)$

*N_τ_, number of considered time windows τ; N_λ_, amount of searching cycles in LASSO regularization terms*.

a *Indicated time per subject*.

Consequently, the proposed enhanced bag-of-patterns representation promotes in motor imagery the following two contributions:

– *Accuracy improvement of bi-conditional tasks*. The effectiveness of conventional CSP extraction is very affected by the time window of EEG segments due to the significant inter- and intra-subject variation. To cope with this issue, we propose to build representations based om bag-of-patterns using the expanded atom-based instances extracted from the spectral-temporal Rayleigh quotient. As a result, the designed dictionary using the higher-level structures allows capturing the structural dynamics of EEG data more carefully over a wide range of τ. Therefore, it increases the classification accuracy, outperforming the baseline sparse CSP-based systems reported in the literature.
– *Better understanding of dynamic brain behavior*. A better understanding of dynamic brain-behavior through the learned LASSO fits. In the designed dictionary of higher-level structures, the model interpretability is increased since the sparse feature selection eliminates irrelevant variables, which are not associated with the response variable. Thus, the learned sparse vector from the bag-of-pattern representations reveals a dynamic behavior that somehow resembles an elicited ERP waveform, rising in the beginning and declining in the closing periods.

However, the Multiple-instance learning algorithms often provide a large number of redundant or irrelevant features, which limits their application for large datasets. Intending to optimize the bag-of-patterns representations, we include a multiple instance Regularization with LASSO penalty and an embedded feature selection that improves further the performed accuracy, increasing the subject performance with a low SNR.

As future work, the authors intend to work out two main issues: computational burden and robustness. To address the former concern, we plan to introduce an instance selection stage, relying on a filter-type measure of performance, like the Rayleigh coefficient. In the latter case, to improve the robustness across trials, the authors are exploring more powerful representations based on bag-of-patterns, using the disgregation/selection of filter-banked components and testing other distances between high-level structures of time series. Further, the computational complexity must be minimized, encouraging validation of the proposed approach on more extensive EEG databases with a higher number of electrodes, multiple labels, and larger populations. Also, we plan to include other motor imagery tasks, extending the present methodology to the multi-class case and applying a set of binary CSP subproblems.

## Data Availability Statement

Publicly available datasets were analyzed in this study. This data can be found here: http://www.bbci.de/competition/iv/#download.

## Author Contributions

DC-H, JC-A, GC-D, and CA-M conceived of the presented idea. DC-H and JC-A developed the theory based on Multiple Instance Learning Representation and performed the computations. GC-D and CA-M verified the analytical methods. DC-H and GC-D encouraged. JC-A to investigate the influence of the selection of time-window in the feature extraction procedures, and supervised the findings of this work. All authors discussed the results and contributed to the final manuscript.

### Conflict of Interest

The authors declare that the research was conducted in the absence of any commercial or financial relationships that could be construed as a potential conflict of interest. The reviewer FG declared a shared affiliation, with no collaboration, with the authors to the handling editor.

## Footnotes

^1^http://www.bbci.de/competition/iv/

## References

[B1] Ang K. K. Chin Z. Y. Wang C. Guan C. Zhang H. (2012). Filter bank common spatial pattern algorithm on BCI competition IV datasets 2a and 2b. Front. Neurosci. 6:39. 10.3389/fnins.2012.00039 22479236 PMC3314883

[B2] Bailly A. Malinowski S. Tavenard R. Guyet T. Chapel L. (2016). Dense bag-of-temporal-SIFT-words for time series classification. CoRR abs/1601.01799. 10.1007/978-3-319-44412-3_2

[B3] Blankertz B. Tomioka R. Lemm S. Kawanabe M. Muller K. (2008). Optimizing spatial filters for robust eeg single-trial analysis. IEEE Signal Process. Magaz. 25, 41–56. 10.1109/MSP.2008.4408441

[B4] Caicedo Acosta J. C. (2019). Time-series representation framework based on multi-instance similarity measures (Master's Thesis). Universidad Nacional de Colombia-Sede Manizales, Manizales, Colombia.

[B5] Caicedo-Acosta J. Cardenas-Pena D. Collazos-Huertas D. Padilla-Buritica J. I. Castano-Duque G. Castellanos-Dominguez G. (2019). Multiple-instance lasso regularization via embedded instance selection for emotion recognition, in Understanding the Brain Function and Emotions, eds Ferrández Vicente J. Álvarez-Sánchez J. de la Paz López F. Toledo Moreo J. Adeli H. (Cham: Springer), 244–251.

[B6] Chen P. Chen C. Yang C. Chang S. Lee K. (2017). MILR: Multiple-instance logistic regression with LASSO penalty. R J. 9, 446–457. 10.32614/RJ-2017-013

[B7] Chen Y. Bi J. Wang J. Z. (2006). MILES: multiple-instance learning via embedded instance selection. IEEE Trans. Pattern Anal. Mach. Intell. 28, 1931–1947. 10.1109/TPAMI.2006.248 17108368

[B8] Cheplygina V. Tax D. Loog M. (2015). Multiple instance learning with bag dissimilarities. Pattern Recogn. 48, 264–275. 10.1016/j.patcog.2014.07.022

[B9] Feng J. K. Jin J. Daly I. Zhou J. Niu Y. Wang X.. (2019). An optimized channel selection method based on multifrequency csp-rank for motor imagery-based bci system. Comp. Int. Neurosci. 2019:8068357. 10.1155/2019/8068357 31214255 PMC6535844

[B10] Fonti V. Belitser E. (2017). Feature selection using LASSO, in Amsterdam Research Paper in Business Analytics (Amsterdam).

[B11] Frau-Meigs D. (2007). Media Education. A Kit for Teachers, Students, Parents and Professionals. UNESCO.

[B12] Gui Z.-W. Yeh Y.-R. (2014). Time series classification with temporal bag-of-words model, in Technologies and Applications of Artificial Intelligence, eds Cheng S.-M. Day M.-Y. (Cham: Springer International Publishing), 145–153.

[B13] Guillot A. Debarnot U. (2019). Benefits of motor imagery for human space flight: a brief review of current knowledge and future applications. Front. Physiol. 10:396. 10.3389/fphys.2019.00396 31031635 PMC6470189

[B14] Liang S. Choi K.-S. Qin J. Pang W.-M. Wang Q. Heng P.-A. (2016). Improving the discrimination of hand motor imagery via virtual reality based visual guidance. Comput. Methods Programs Biomed. 132, 63–74. 10.1016/j.cmpb.2016.04.023 27282228

[B15] Lin J. Li Y. (2009). Finding structural similarity in time series data using bag-of-patterns representation, in Scientific and Statistical Database Management, ed Winslett M. (Berlin; Heidelberg: Springer), 461–477.

[B16] Miao M. Wang A. Liu F. (2017). A spatial-frequency-temporal optimized feature sparse representation-based classification method for motor imagery EEG pattern recognition. Med. Biol. Eng. Comput. 55, 1589–1603. 10.1007/s11517-017-1622-1 28161876

[B17] Padfield N. Zabalza J. Zhao H. Vargas V. Ren J. (2019). EEG-based brain-computer interfaces using Motor-Imagery: techniques and challenges. Sensors 19, 1–34. 10.3390/s19061423 30909489 PMC6471241

[B18] Padilla-Buritica J. Hurtado J. Castellanos-Dominguez G. (2019). Supervised piecewise network connectivity analysis for enhanced confidence of auditory oddball tasks. Biomed. Signal Process. Control 52, 341–346. 10.1016/j.bspc.2019.04.020

[B19] Passalis N. Tsantekidis A. Tefas A. Kanniainen J. Gabbouj M. Iosifidis A. (2017). Time-series classification using neural Bag-of-Features, in 2017 25th European Signal Processing Conference (EUSIPCO) (Kos), 301–305.

[B20] Pfannschmidt L. Jakob J. Biehl M. Tino P. Hammer B. (2019). Feature Relevance Bounds for Ordinal Regression. CoRR abs/1902.07662.

[B21] Ratanamahatana C. A. Keogh E. (2004). Making time-series classification more accurate using learned constraints, in Proceedings of the 2004 SIAM International Conference on Data Mining (Lake Buena Vista, FL), 11–22.

[B22] Roth V. (2004). The generalized LASSO. IEEE Trans. Neural Netw. 15, 16–28. 10.1109/TNN.2003.809398 15387244

[B23] Shin Y. Lee S. Lee J. Lee H.-N. (2012). Sparse representation-based classification scheme for motor imagery-based brain–computer interface systems. J. Neural Eng. 9:056002. 10.1088/1741-2560/9/5/056002 22872668

[B24] Suica Z. Platteau-Waldmeier P. Koppel S. Schmidt-Trucksaess A. Ettlin T. Schuster-Amft C. (2018). Motor imagery ability assessments in four disciplines: protocol for a systematic review. BMJ Open 8:e023439. 10.1136/bmjopen-2018-023439 30552265 PMC6303557

[B25] Wang J. Liu P. She M. Nahavandi S. Kouzani A. (2013). Bag-of-words representation for biomedical time series classification. Biomed. Signal Process. Control 8, 634–644. 10.1016/j.bspc.2013.06.004

[B26] Wang K. Xu M. Wang Y. Zhang S. Chen L. Ming D. (2019). Enhance decoding of pre-movement eeg patterns for brain-computer interfaces. J. Neural Eng. 17:016033. 10.1088/1741-2552/ab598f 31747642

[B27] Xu Y. Zhang Z. Yang J. Li X. Zhang D. (2015). A survey of sparse representation: algorithms and applications. IEEE Access 3, 490–530. 10.1109/ACCESS.2015.2430359

[B28] Yamawaki N. Wilke C. Liu Z. He B. (2006). An enhanced time-frequency-spatial approach for motor imagery classification. IEEE Trans. Neural Syst. Rehabil. Eng. 14, 250–254. 10.1109/TNSRE.2006.875567 16792306 PMC1989674

[B29] Zhang Y. Nam C. S. Zhou G. Jin J. Wang X. Cichocki A. (2018). Temporally constrained sparse group spatial patterns for motor imagery BCI. IEEE Trans. Cybernet. 49, 1–11. 10.1109/TCYB.2018.2841847 29994667

[B30] Zhang Y. Zhou G. Jin J. Wang X. Cichocki A. (2015). Optimizing spatial patterns with sparse filter bands for motor-imagery based brain-computer interface. J. Neurosci. Methods 255, 85–91. 10.1016/j.jneumeth.2015.08.004 26277421

